# Characteristics of Diagnosed and Death Cases of Pneumoconiosis in Hubei Province, China, 1949–2019

**DOI:** 10.3390/ijerph192315799

**Published:** 2022-11-27

**Authors:** Yuxin Yao, Tingting Wei, Hai Zhang, Yujia Xie, Pei Gu, Yongxiang Yao, Xin Xiong, Zhe Peng, Zhong Zhen, Sheng Liu, Xiuqing Cui, Liangying Mei, Jixuan Ma

**Affiliations:** 1 Department of Occupational and Environmental Health, School of Public Health, Tongji Medical College, Huazhong University of Science and Technology, Wuhan 430030, China; 2 Key Laboratory of Environment and Health, Ministry of Education and Ministry of Environmental Protection, and State Key Laboratory of Environmental Health (Incubating), School of Public Health, Tongji Medical College, Huazhong University of Science and Technology, Wuhan 430030, China; 3 Institute of Health Surveillance, Analysis and Protection, Center for Disease Control and Prevention of Hubei Province, Wuhan 430030, China

**Keywords:** pneumoconiosis, diagnosed, death, retrospective study

## Abstract

Objective: This study aims to summarize the characteristics of diagnosed pneumoconiosis and pneumoconiosis death in the Hubei Province of China, between the years 1949 and 2019, and provide clues for the scientific prevention of pneumoconiosis. Methods: We recruited 23,069 pneumoconiosis cases in Hubei Province, China, from 1949 to 2019. Basic information and occupational surveillance information were obtained from the Hubei Occupational Diseases and Health Risk Factors Information Surveillance System. Results: The annually diagnosed pneumoconiosis cases showed an overall increasing trend from 1949 to 2019 in Hubei Province. The major types of pneumoconiosis were coal workers’ pneumoconiosis (CWP, 49.91%) and silicosis (43.39%). Pneumoconiosis cases were mainly engaged in mining (75.32%) and manufacturing (12.72%), and were distributed in Huangshi (35.48%), Yichang (16.16%), and Jingzhou (7.97%). CWP (47.50%) and silicosis (44.65%) accounted for most of the deaths. Conclusions: The number of pneumoconiosis cases and deaths in Hubei increased in the period of 1949 to 2019. Silicosis and CWP contributed to the predominant types of pneumoconiosis. Prevention and control measures should continue to be taken to reduce the morbidity and mortality of pneumoconiosis.

## 1. Introduction

Pneumoconiosis represents a series of interstitial lung diseases, including silicosis and coal workers’ pneumoconiosis (CWP), due to the inhalation of occupational respirable dust, such as silica dust, coal mine dust, or asbestos fibers [1]. Occupational respirable dust is generated during the mining, drilling, cutting, polishing, and grinding of sandstone or marble, or during the crushing and sieving process of raw materials in manufacturing [2]. When the dust enters and accumulates in the lung tissues, chronic inflammation and pulmonary fibrosis may occur, causing irreversible damage to the lungs [3]. More importantly, workers exposed to occupational respirable dust were often asymptomatic in the early stages, while the diseases could worsen even after the workers leave the dust-exposed environment [4].

The global incidence of pneumoconiosis is still increasing to date. According to the Global Burden of Disease Study, the incidence of pneumoconiosis rose from 36,186 in 1990 to 60,055 in 2017 [5], the number of deaths from pneumoconiosis increased from 21,209 in 1990 to 21,488 in 2016, and disability-adjusted life-years (DALYs) increased from 567,941 to 576,977 [6]. Geographically, Southeast Asia, East Asia, and Oceania had the highest prevalence of pneumoconiosis [7]. It was worth noting that more than half of the newly diagnosed pneumoconiosis cases worldwide were found in China (32,205/60,055 cases) [5]. Data from the National Health Commission of China suggested that there were 15,407 occupational disease events in 2021, of which 76.65% (11,809 cases) were pneumoconiosis [8]. However, previous studies further suggested that the actual burden of pneumoconiosis may be underestimated due to the low frequency of occupational health checks and inadequate reporting systems [1,9]. Accurate descriptions of the long-term characteristics of pneumoconiosis are required and this may provide clues to the prevention and management of pneumoconiosis.

Therefore, we conducted the present study to update the data on pneumoconiosis in Hubei, China, from 1949 to 2019. This study aims to summarize the characteristics and distribution of pneumoconiosis and to analyze the composition of causes of death.

## 2. Materials and Methods

### 2.1. Study Subjects

A retrospective survey of occupational pneumoconiosis, diagnosed between 1949 and 2019, was conducted in Hubei Province, from June 2019 to August 2020. We recruited a total of 24,753 pneumoconiosis cases diagnosed between 1 January 1949 and 31 December 2019 in Hubei Province, China into the analysis. Demographic and occupational exposure history information were obtained from the Hubei Occupational Diseases and Health Risk Factors Information Surveillance System. Data on death status was obtained from the Coroner Surveillance System. After excluding subjects with missing data on diagnostic information (*n* = 1684), a total of 23,069 cases were included in the final analysis.

### 2.2. Diagnosis of Pneumoconiosis

According to the X-ray staging and diagnostic criteria of silicosis (1963), Chinese X-ray diagnosis of pneumoconiosis (GB5906-86, GB5906-97), and Chinese diagnosis criteria of occupational pneumoconiosis (GBZ70-2002, GBZ70-2009, GBZ70-2015), pneumoconiosis was classified into 13 categories including silicosis, CWP, asbestosis, cement pneumoconiosis, welder pneumoconiosis, founder pneumoconiosis, graphite pneumoconiosis, carbon black pneumoconiosis, talc pneumoconiosis, mica pneumoconiosis, kaolin pneumoconiosis, aluminosis, and other types of pneumoconiosis. The diagnosis and definition of pneumoconiosis were in accordance with the Diagnosis of Occupational pneumoconiosis (GBZ70-2015) and the Pathological diagnosis criteria of pneumoconiosis (GBZ25-2014).

### 2.3. Industrial Classification

The industrial classification was divided into 20 categories, according to the Chinese Industrial Classification for National Economic Activities (GB/T4754-2017). In this study, we further categorized industries with fewer cases as “Other types” to better illustrate the results. The “Other types” include electricity, heat, gas and water production and supply; wholesale and retail industry; transportation, warehousing and postal services industry; hotel and restaurant industry; information transmission, software and technology services industry; financial industry; real estate industry; leasing and business services industry; scientific research and technology services industry; water conservancy, environment and public facilities management industry; residential services, repairs and other services industry; education industry; health and social work industry; culture, sports and entertainment industry; and international organization. In summary, the industries in this study were presented as farming/forestry/husbandry/fishery, mining, manufacturing, construction, public management/social security/social organization, and other types of industries.

### 2.4. Determination of Cause of Death

Fundamental causes were coded according to the Classification and codes of diseases (GB/T14396-2016), which was a Chinese standard based on the International Classification of Diseases, 10th revision (ICD-10), for expansion and refinement.

### 2.5. Statistical Analysis

The basic characteristics of pneumoconiosis cases were reported as mean (standard deviation, SD) or median (interquartile range, IQR) for continuous variables, and as number (percentage) for categorical variables. The Chi-square test was used for group comparisons of categorical variables, and the One-way ANOVA or Kruskal-Wallis rank-sum test was used for comparisons of continuous variables between groups. All *p*-values were two-sided with a significant level of 0.05, and statistical analyses were conducted using R software (version 4.1.3, R Core Team, Vienna, Austria).

## 3. Results

### 3.1. Pneumoconiosis Cases in Hubei Province from 1949 to 2019

**Figure 1:**
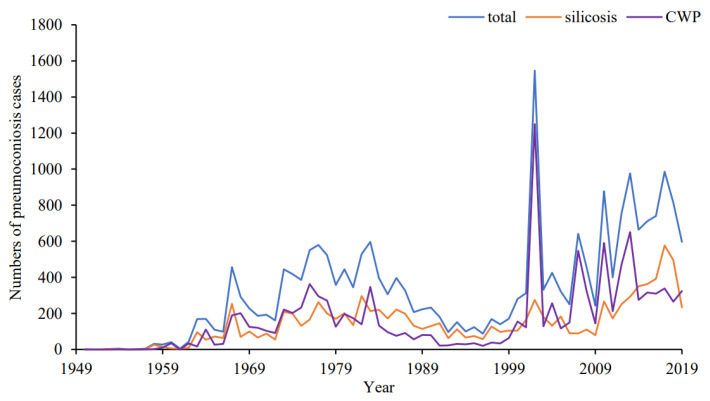
Numbers of pneumoconiosis cases in Hubei province, China, 1949–2019.

As shown in Figure 1, the annually diagnosed pneumoconiosis cases showed an overall increasing trend, fluctuating between 1949 and 1983, reaching a maximum of 596 in 1983, gradually decreasing to 88 in 1996, and then increasing rapidly in the next six years (1996–2002), peaked in 2002 with 1546 cases. Among the 23,069 pneumoconiosis cases, 17,315 (75.06%) were in stage I, 4253 (18.44%) in stage II, and 1501 (6.50%) in stage III. The majority of the cases were of male patients (22,599 cases, 97.96%). The number (percent) of silicosis and CWP were 10,010 (43.39%) and 11,513 (49.91%) cases, respectively. A total of 17,043 (73.88%) cases had been exposed to dust for over ten years (Table 1).

### 3.2. Industrial Difference between Pneumoconiosis Cases

A total of 17,376 (75.32%) pneumoconiosis cases were distributed in the mining industry, and the manufacturing industry ranked second (2935 cases, 12.72%). The silicosis cases mainly occurred in the mining and manufacturing industries, accounting for 58.58% (5864 cases) and 20.70% (2072 cases), respectively. The CWP cases were almost entirely concentrated in the mining industry (11,039 cases, 95.88%). The cement pneumoconiosis cases were mainly in the construction industry (230 cases, 50.11%) and the manufacturing industry (153 cases, 33.33%). Both welder pneumoconiosis and founder pneumoconiosis were mostly in the manufacturing industry, with 106 cases (79.70%) and 282 cases (93.69%), respectively (Table 2).

### 3.3. District Distribution among Pneumoconiosis Cases

**Figure 2:**
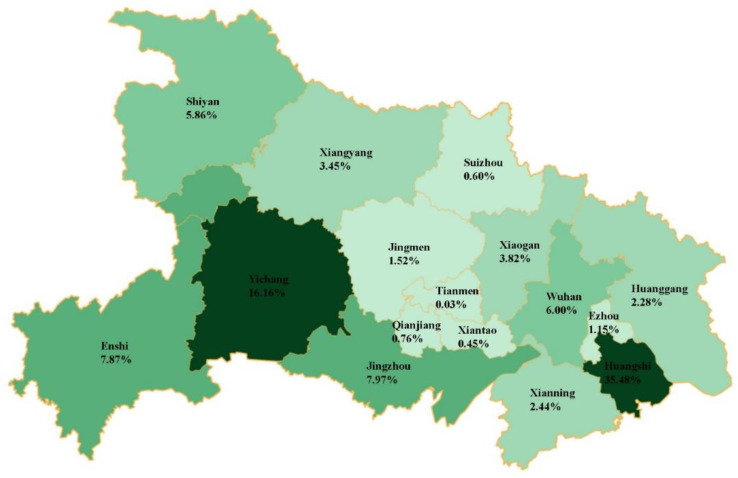
The Regional Distribution of Pneumoconiosis Cases Reported in Hubei, 1949–2019.

The top three cities reporting pneumoconiosis in Hubei Province were Huangshi (8184 cases, 35.48%), Yichang (3728 cases, 16.16%), and Jingzhou (1839 cases, 7.97%). Silicosis cases were mainly distributed in Huangshi (2614 cases, 26.11%), Wuhan (1196 cases, 11.95%), and Jingzhou (1105 cases, 11.04%). Approximately half of the CWP cases were in Huangshi (5314 cases, 46.15%), while Yichang (2595 cases, 22.54%) and Enshi (1568 cases, 13.62%) ranked second and third in CWP cases, respectively. Cement pneumoconiosis cases were predominantly in Huangshi (226 cases, 49.24%), while welder pneumoconiosis and founder pneumoconiosis cases were both mainly in Shiyan, with 33 cases (24.81%) and 137 cases (45.52%), respectively (Figure 2 and Table 3).

### 3.4. Characteristics Information in Deaths and Survivors of Pneumoconiosis

Among the 23,069 pneumoconiosis cases, 12,076 (52.35%) of the patients survived and 10,993 (47.65%) resulted in death. The distribution of sex, types of pneumoconiosis, exposure duration, and age of onset showed a significant difference between survival and death cases (*p* < 0.001). The proportion of females in the death group (2.71%) was higher than that in the survival group (1.42%) (*p* < 0.001). Silicosis and CWP were predominant in both survival and death groups. Among the survivors, 68.61% of the cases were exposed to dust ≥10 years, and this percentage increased to 79.67% among the deaths (*p* < 0.001). In addition, welder pneumoconiosis showed a lower proportion of death cases (12/133 cases, 9.02%) than any other type (Table 4).

The number of pneumoconiosis deaths increased monotonically from 1958 to 1988 and continued to increase, in a fluctuating pattern, between 1989 to 2019, reaching 422 deaths in 2019 (Figure S1). We further classified the causes of death into 15 categories based on ICD-10. Among all the categories of causes, pneumoconiosis was the leading cause of death (600 cases, 5.46%), followed by cardiovascular and cerebrovascular diseases (485 cases, 4.41%). Lung tumors and malignant tumors in other systems were another important cause of death, with 209 cases (1.90%) and 249 cases (2.27%), respectively (Table S1).

## 4. Discussion

This study reported the characteristics of 23,069 cases of pneumoconiosis in Hubei Province from 1949 to 2019. The number of annually diagnosed pneumoconiosis cases in Hubei Province showed an overall upward trend. The major types of pneumoconiosis were CWP and silicosis, and the cases were mainly engaged in mining and manufacturing, with the highest number of cases in Huangshi, Yichang and Jingzhou. Pneumoconiosis, as well as cardiovascular and cerebrovascular diseases, were the main causes of death. Our results presented the distribution pattern of pneumoconiosis in Hubei Province, which may provide clues for the prevention and management of pneumoconiosis.

In this study, we found that new cases of pneumoconiosis have been increasing, in a fluctuating pattern, from 1949 to 2019, with similar trends for CWP and silicosis. These results are consistent with previous studies in China [10,11,12]. A possible explanation for the increasing trend may be the rapid economic development of Hubei and the demand for coal energy. Between 1949 and 1959, only a few cases were reported, probably due to the incomplete reporting system. The promulgation of the Law of the People’s Republic of China on Prevention and Control of Occupational Diseases in 2002 may have contributed to stronger supervision measures, therefore the greatest number of new cases was observed in the same year. The increase in new cases each year since 2006 might be due to the establishment of the Network Direct Report System of Occupational diseases in 2006, which has made case reporting more comprehensive and convenient [4].

CWP was the predominant type of pneumoconiosis in Hubei, followed by silicosis, which was consistent with previous studies conducted in other provinces and cities in China [9,13,14]. For example, Nie et al. reported that CWP and silicosis accounted for 67.00% and 30.46% of 7312 cases in Hunan Province from 2006 to 2010, respectively [9]. Furthermore, Sun et al. found that the percentage of CWP in pneumoconiosis in Heilongjiang Province was 87.18% between 2006 and 2018 [15]. However, other researchers found that silicosis was the dominant type of pneumoconiosis in other Chinese cities [16,17,18]. In the UK, researchers found that there were 1070 cases of pneumoconiosis diagnosed in the UK between 1997 and 2008, of which 78.50% were asbestosis [19]. Erol et al. also reported that the incidence of CWP in Turkey has been approximately six times higher than that in the USA over the past decade, which indicated that CWP remained the most prevalent occupational health issue in Turkey [20]. These inconsistent results may be due to the different industrial structures and economic types in each province and country. For example, Hubei and Heilongjiang provinces have many coal mines and a higher number of workers exposed to coal dust than other cities in China; therefore, they had a higher incidence of CWP [11]. Hebei Province is dominated by steel, coal, petrochemical and manufacturing industries; thus, it reported a higher incidence of silicosis in China [12]. A previous study also reported that in 2010, pneumoconiosis remained the main type of Chinese coal miners’ occupational disease, while it only accounted for 26% of the USA coal miners’ occupational disease, which may be explained by the great differences in technology between countries. For example, the USA government has promoted the use of new ventilation systems; therefore, the incidence of pneumoconiosis and the proportion it accounted for occupational diseases were low [21]. Taken together, our findings indicated that silicosis and CWP remain the most serious occupational diseases in China, and it is necessary to take implement to control the concentrations of dust in workplaces.

For the duration of dust exposure, 73.88% of pneumoconiosis cases had been exposed to dust for more than ten years, indicating that chronic pneumoconiosis remains the predominant type. This finding was similar to previous studies; for example, Xia et al. observed that 66.68% of pneumoconiosis cases diagnosed between 2008 and 2013 had more than ten years of dust exposure [22]. In this study, we found that silicosis and CWP were the main types of pneumoconiosis in both groups with less and more than ten years of dust exposure. This difference may be due to the different pathogenic hazard capacity of each dust, for example, silica dust has a higher toxicity than coal dust, therefore silicosis usually develops faster than CWP [23]. Differences in the length of exposure may also be explained by differences in daily working hours, dust concentration, and dust protection in different working environments [24].

Stage I pneumoconiosis accounted for the majority of pneumoconiosis cases, similar to the national report in China [13]. The high proportion of stage I pneumoconiosis may reflect the effectiveness of the control measures taken in China in recent years [25]. In contrast to past limitations, regular screening now allows us to detect cases at an early stage, which is more beneficial to the control and treatment of the occurrence and development of pneumoconiosis. Strengthened monitoring of occupational diseases would also be helpful for the early detection, diagnosis, and treatment of pneumoconiosis.

Compared with survival cases, death cases had a significantly higher exposure duration, which supported previous findings that long-term exposure to occupational dust may be associated with increased mortality risk [26,27,28]. Consistent with previous findings [29,30], our study found that pneumoconiosis was the leading cause of death, followed by cardiovascular and cerebrovascular diseases, tumors, and pneumoconiosis-related complications such as pulmonary heart disease. Su et al. reported that respiratory diseases accounted for 70.12% of pneumoconiosis deaths in Henan Province [31]. Pneumoconiosis was the leading cause of death, most likely due to the irreversible and progressive features of pulmonary fibrosis, which leads to a continuous decline in respiratory function or impaired self-purification of the patient’s respiratory system. Combined with reduced immune function, pneumoconiosis patients are vulnerable to dying from lung and bronchial infections [29]. Similar to a previous finding [32], we found that silicosis and CWP accounted for 92.15% of the total number of deaths. Therefore, it would be of great significance to public health to strengthen the prevention of silicosis and CWP, slow down the progression of the diseases, and prolong the survival time of pneumoconiosis patients.

Our research has several strengths. First, the large sample size and long observation period render our results reliable and stable. Second, our results could provide insight into the distribution characteristics of pneumoconiosis. However, our study also has several limitations. First, the observational study could not assess the causal relationship between dust exposure and pneumoconiosis, nor could it change the understanding of the pathology of pneumoconiosis. Second, the data from the surveillance system may have reporting bias. In addition, the data may be affected by the uncertainties of the surveillance system, particularly in the early years. However, our data were obtained by aggregating data from the Hubei Occupational Diseases and Health Risk Factors Information Surveillance System; therefore, the data were in a standard format and relatively complete. Third, we didn’t collect data on the number of workers exposed to dust each year, thus we cannot calculate the relative risk of pneumoconiosis for each working sector. However, our study found an increasing trend of new cases of pneumoconiosis and that the number of cases was closely related to the distribution of minerals and the type of industry. Our results highlighted silicosis and CWP as the types of pneumoconiosis that require priority management, and that the mining and manufacturing industries require priority surveillance. This study also highlighted the importance of improving the work environment and strengthening occupational surveillance in key industries.

## 5. Conclusions

Our study found that the number of pneumoconiosis cases and deaths in Hubei Province, China, have remained high over the past 70 years. Silicosis and CWP were the predominant types and were mainly engaged in mining and manufacturing. The proportion of cases was much higher in the ≥10 years exposure duration group, and the leading cause of death was pneumoconiosis. Our findings provided an accurate description of the long-term characteristics of pneumoconiosis in Hubei Province and can help to understand the current status of pneumoconiosis, highlight the priority of reducing silicosis and CWP cases, and provide clues for the prevention and management of pneumoconiosis.

## Figures and Tables

**Table 1:** Basic Characteristics of Pneumoconiosis Patients in Hubei Province, 1949–2019.

Variables	Types of Pneumoconiosis							*p*
	Total (*n* = 23,069)	Silicosis (*n* = 10,010)	Coal Workers’ Pneumoconiosis (*n* = 11,513)	Cement Pneumoconiosis (*n* = 459)	Welder Pneumoconiosis (*n* = 133)	Founder Pneumoconiosis (*n* = 301)	Other Types of Pneumoconiosis ^a^ (*n* = 653)‫	
Sex, *n* (%)								<0.001
Male	22,599 (97.96)	9669 (42.78)	11,480 (50.80)	424 (1.88)	126 (0.56)	287 (1.27)	613 (2.71)	
Female	470 (2.04)	341 (72.55)	33 (7.02)	35 (7.45)	7 (1.49)	14 (2.98)	40 (8.51)	
Exposure Duration, *n* (%), Years								<0.001
<10	5192 (22.51)	3111 (59.92)	1825 (35.15)	66 (1.27)	52 (1.00)	42 (0.81)	96 (1.85)	
≥10	17,043 (73.88)	6508 (38.19)	9643 (56.58)	368 (2.16)	79 (0.46)	227 (1.33)	218 (1.28)	
Unknown	834 (3.61)	391 (46.88)	45 (5.40)	25 (3.00)	2 (0.24)	32 (3.83)	339 (40.65)	
First Stage, *n* (%)								<0.001
Stage I	17,315 (75.06)	7305 (42.19)	8691 (50.19)	423 (2.44)	115 (0.66)	285 (1.65)	496 (2.87)	
Stage II	4253 (18.44)	1962 (46.13)	2092 (49.19)	27 (0.63)	16 (0.38)	13 (0.31)	143 (3.36)	
Stage III	1501 (6.50)	743 (49.50)	730 (48.64)	9 (0.60)	2 (0.13)	3 (0.20)	14 (0.93)	
Newly Diagnosed Patients, *n* (%)								<0.001
1949–1959	72 (0.31)	46 (63.89)	15 (20.83)	6 (8.33)	— ^b^	—	5 (6.95)	
1959–1969	1609 (6.97)	726 (45.12)	765 (47.55)	8 (0.50)	2 (0.12)	18 (1.12)	90 (5.59)	
1969–1979	3800 (16.47)	1547 (40.71)	2024 (53.26)	74 (1.95)	1 (0.03)	28 (0.74)	126 (3.31)	
1979–1989	3769 (16.34)	1902 (50.46)	1387 (36.80)	145 (3.85)	13 (0.34)	165 (4.38)	157 (4.17)	
1989–1999	1451 (6.29)	974 (67.13)	371 (25.56)	50 (3.45)	3 (0.21)	18 (1.24)	35 (2.41)	
1999–2009	4806 (20.83)	1407 (29.28)	3193 (66.44)	90 (1.87)	10 (0.21)	29 (0.60)	77 (1.60)	
2009–2019	7517 (32.59)	3390 (45.10)	3749 (49.87)	86 (1.14)	104 (1.39)	43 (0.57)	145 (1.93)	
Unknown	45 (0.20)	18 (40.00)	9 (20.00)	—	—	—	18 (40.00)	

^a^ Other types of pneumoconiosis include: graphite pneumoconiosis, carbon black pneumoconiosis, asbestosis, talc pneumoconiosis, pottery worker’s pneumoconiosis, aluminosis, other pneumoconiosis, pneumoconiosis (unknown). ^b^—represents a value of 0.

**Table 2:** Industry Distribution in Different Types of Pneumoconiosis Cases.

Industry	Types of Pneumoconiosis, *n* (%)							*p*
	Total (*n* = 23,069)	Silicosis (*n* = 10,010)	Coal Workers’ Pneumoconiosis (*n* = 11,513)	Cement Pneumoconiosis (*n* = 459)	Welder Pneumoconiosis (*n* = 133)	Founder Pneumoconiosis (*n* = 301)	Other Types of Pneumoconiosis ^a^ (*n* = 653)	
Mining industry	17,376 (75.32)	5864 (58.58)	11,039 (95.88)	62 (13.51)	14 (10.53)	8 (2.66)	389 (59.57)	<0.001
Manufacturing	2935 (12.72)	2072 (20.70)	111 (0.96)	153 (33.33)	106 (79.70)	282 (93.69)	211 (32.31)	
Public management, social security and social organization	1240 (5.38)	980 (9.79)	253 (2.20)	1 (0.22)	1 (0.75)	3 (0.99)	2 (0.31)	
Construction industry	896 (3.89)	625 (6.24)	16 (0.14)	230 (50.11)	2 (1.50)	1 (0.33)	22 (3.37)	
Farming, forestry, husbandry and fishery	72 (0.31)	37 (0.37)	31 (0.27)	— ^b^	—	—	4 (0.61)	
Other types of industries	550 (2.38)	432 (4.32)	63 (0.55)	13 (2.83)	10 (7.52)	7 (2.33)	25 (3.83)	

^a^ Other types of pneumoconiosis include: graphite pneumoconiosis, carbon black pneumoconiosis, asbestosis, talc pneumoconiosis, pottery worker’s pneumoconiosis, aluminosis, other pneumoconiosis, pneumoconiosis (unknown). ^b^—represents a value of 0.

**Table 3:** District Distribution in Different Types of Pneumoconiosis Cases.

District	Types of Pneumoconiosis, *n* (%)							*p*
	Total (*n* = 23,069)	Silicosis (*n* = 10,010)	Coal Workers’ Pneumoconiosis (*n* = 11,513)	Cement Pneumoconiosis (*n* = 459)	Welder Pneumoconiosis (*n* = 133)	Founder Pneumoconiosis (*n* = 301)	Other Types of Pneumoconiosis ^a^ (*n* = 653)	
Huangshi	8184 (35.48)	2614 (26.11)	5314 (46.15)	226 (49.24)	7 (5.26)	1 (0.33)	22 (3.37)	<0.001
Yichang	3728 (16.16)	1024 (10.23)	2595 (22.54)	23 (5.01)	22 (16.54)	1 (0.33)	63 (9.65)	
Jingzhou	1839 (7.97)	1105 (11.04)	679 (5.90)	30 (6.53)	8 (6.02)	10 (3.32)	7 (1.07)	
Enshi	1817 (7.87)	213 (2.13)	1568 (13.62)	17 (3.7)	3 (2.26)	1 (0.33)	15 (2.3)	
Wuhan	1383 (6.00)	1196 (11.95)	61 (0.53)	26 (5.66)	5 (3.76)	57 (18.94)	38 (5.82)	
Shiyan	1351 (5.86)	860 (8.59)	276 (2.40)	14 (3.05)	33 (24.81)	137 (45.52)	31 (4.75)	
Xiaogan	882 (3.82)	574 (5.73)	2 (0.02)	1 (0.22)	— ^b^	1 (0.33)	304 (46.56)	
Xiangyang	796 (3.45)	507 (5.06)	97 (0.84)	56 (12.2)	9 (6.77)	72 (23.93)	55 (8.42)	
Xianning	562 (2.44)	340 (3.40)	198 (1.72)	19 (4.14)	2 (1.5)	2 (0.66)	1 (0.15)	
Huanggang	526 (2.28)	132 (1.32)	329 (2.86)	21 (4.58)	1 (0.75)	4 (1.33)	39 (5.97)	
Jingmen	351 (1.52)	239 (2.39)	97 (0.84)	6 (1.31)	1 (0.75)	1 (0.33)	7 (1.07)	
Ezhou	266 (1.15)	246 (2.46)	18 (0.16)	1 (0.22)	—	—	1 (0.15)	
Qianjiang	175 (0.76)	174 (1.74)	—	—	—	1 (0.33)	—	
Suizhou	139 (0.60)	125 (1.25)	5 (0.04)	1 (0.22)	6 (4.51)	2 (0.66)	—	
Xiantao	104 (0.45)	104 (1.04)	—	—	—	—	—	
Tianmen	7 (0.03)	7 (0.07)	—	—	—	—	—	
Unknown ^c^	959 (4.16)	550 (5.49)	274 (2.38)	18 (3.92)	36 (27.07)	11 (3.66)	70 (10.72)	

^a^ Other types of pneumoconiosis include: graphite pneumoconiosis, carbon black pneumoconiosis, asbestosis, talc pneumoconiosis, pottery worker’s pneumoconiosis, aluminosis, other pneumoconiosis, pneumoconiosis (unknown). ^b^—represents a value of 0. ^c^ Distribution information was not detected in the system.

**Table 4:** Characteristics information in Dead and Survival Pneumoconiosis Patients in Hubei, 1949–2019.

Variables	Total (*n* = 23,069)	Survival (*n* = 12,076)	Death (*n* = 10,993)	*p*
Sex, *n* (%)				<0.001
Male	22,599 (97.96)	11,904 (98.58)	10,695 (97.29)	
Female	470 (2.04)	172 (1.42)	298 (2.71)	
Types, *n* (%)				<0.001
Coal workers’ pneumoconiosis	11,513 (49.91)	6291 (52.10)	5222 (47.50)	
Silicosis	10,010 (43.39)	5102 (42.25)	4908 (44.65)	
Cement pneumoconiosis	459 (1.99)	229 (1.89)	230 (2.09)	
Founder pneumoconiosis	301 (1.30)	112 (0.93)	189 (1.72)	
Welder pneumoconiosis	133 (0.58)	121 (1.00)	12 (0.11)	
Other types of pneumoconiosis ^a^	653 (2.83)	221 (1.83)	432 (3.93)	
Exposure Duration, *n* (%), years				<0.001
<10	5192 (22.51)	3750 (31.05)	1442 (13.12)	
≥10	17,043 (73.88)	8285 (68.61)	8758 (79.67)	
Unknown ^b^	834 (3.61)	41 (0.34)	793 (7.21)	
Age of onset, years, mean (SD)	15,342 (66.50)	49.96 (10.23)	53.24 (12.58)	<0.001
Unknown, *n* (%)	7727 (33.50)	12 (0.10)	7715 (70.18)	

^a^ Other types of pneumoconiosis include: graphite pneumoconiosis, carbon black pneumoconiosis, asbestosis, talc pneumoconiosis, pottery worker’s pneumoconiosis, aluminosis, other pneumoconiosis, pneumoconiosis (unknown). ^b^ Information was not detected in the system.

## Data Availability

Not applicable.

## Supplementary Materials

The following supporting information can be downloaded at: https://www.mdpi.com/article/10.3390/ijerph192315799/s1, Figure S1: Numbers of pneumoconiosis deaths in Hubei province, China, 1949–2019; Table S1: Causes of Death in Pneumoconiosis Cases and Corresponding ICDs (*n* = 23,069).
