# Supplementary material for: Characteristics of Diagnosed and Death Cases of Pneumoconiosis in Hubei Province, China, 1949–2019
Source: Int J Environ Res Public Health. 2022 Nov 27;19(23):15799. doi: 10.3390/ijerph192315799 (PMC9740043; doi:10.3390/ijerph192315799)
Supplement: Supplementary file 1 [file ijerph-19-15799-s001.zip › ijerph-2050339-supplementary.pdf]

SUPPLEMENTAL MATERIAL

Characteristics of Diagnosed and Death Cases of Pneumoconiosis in Hubei Province, China, 1949-2019

Yuxin Yao [1,2,†], Tingting Wei [3,†], Hai Zhang [3], Yujia Xie [1,2], Pei Gu [1,2], Yongxiang Yao [3], Xin Xiong [3], Zhe Peng [3], Zhong Zhen [3], Sheng Liu [3], Xiuqing Cui [3], Liangying Mei [3,*] and Jixuan Ma [1,2,*]

1 Department of Occupational and Environmental Health, School of Public Health, Tongji Medical College, Huazhong University of Science and Technology, Wuhan 430030, China
2 Key Laboratory of Environment and Health, Ministry of Education and Ministry of Environmental Protection, and State Key Laboratory of Environmental Health (Incubating), School of Public Health, Tongji Medical College, Huazhong University of Science and Technology, Wuhan 430030, China
3 Institute of Health Surveillance, Analysis and Protection, Center for Disease Control and Prevention of Hubei Province, Wuhan 430030, China
* Correspondence: hbcdcmly@126.com (L.M.); maj@hust.edu.cn (J.M.)
† These authors contributed equally to this work.

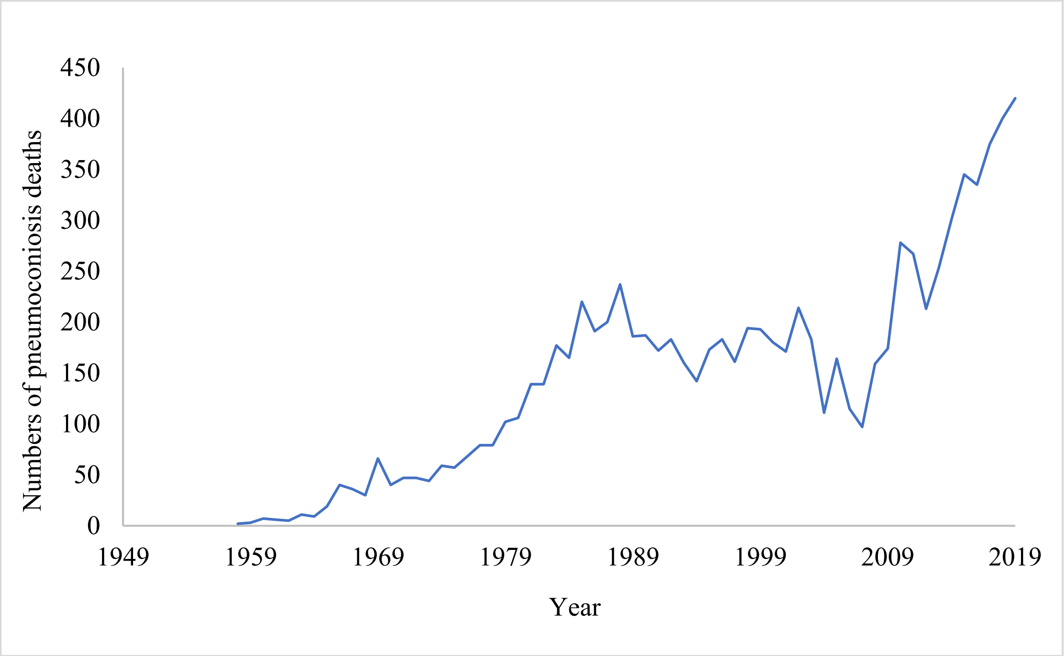

Figure S1. Numbers of pneumoconiosis deaths in Hubei province, China, 1949-2019.

Table S1. Causes of Death in Pneumoconiosis Cases and Corresponding ICDs (n = 10,993).

Cause of Death	ICDs	Number
Tuberculosis	A15-A16, B90	15
Diseases of other systems	A41, B18-B24, B94, E10-E14, E85, F03, F10, G30-G93, I12-I13, I72-I99, K25-K92, M06-N32, P10, R0-R65, T28, T60	112
Other malignant tumors	C04-C96, D00-D80	249
Tumors in the lung	C34, C38, D02, K74	209
Cardiovascular and cerebrovascular diseases	I00-I11, I14-I25, I28-I71	485
Pulmonary heart disease	I26-I27	36
Acute respiratory infection	J00-J22	15
Chronic respiratory infection	J41-J42	8
Emphysema	J43, J98	27
Chronic obstructive pulmonary disease	J44	164
Other lung diseases	J45-J98, R06	21
Pneumoconiosis	J60–J65	600
Respiratory failure	J96	35
Unexplained death	R98-R99, Y34, Y86	8,959
Injury	V01-X80	58

Abbreviation: ICD-10-CM, International Classification of Diseases, Tenth Revision, Clinical Modification.
